# Time-varying effect of postoperative cholesterol profile on long-term outcomes of isolated coronary artery bypass graft surgery

**DOI:** 10.1186/s12944-023-01927-8

**Published:** 2023-10-03

**Authors:** Aryan Ayati, Kasra Akbari, Akbar Shafiee, Arezou Zoroufian, Arash Jalali, Sahar Samimi, Mina Pashang, Kaveh Hosseini, Jamshid Bagheri, Farzad Masoudkabir

**Affiliations:** 1 Tehran Heart Center, Cardiovascular Diseases Research Institute, Tehran University of Medical Sciences, Tehran, Iran; 2 Cardiac Primary Prevention Research Center, Cardiovascular Diseases Research Institute, Tehran University of Medical Sciences, Tehran, Iran; 3 Department of Epidemiology and Biostatistics, School of Public Health, Tehran University of Medical Sciences, Tehran, Iran; 4 Research Center for Advanced Technologies in Cardiovascular Medicine, Cardiovascular Diseases Research Institute, Tehran University of Medical Sciences, Tehran, Iran

**Keywords:** Low-density lipoprotein to high-density lipoprotein ratio, Cholesterol profile, Coronary artery disease, Coronary artery bypass graft surgery, Time-varying covariates, Competing risk, LDL/HDL ratio

## Abstract

**Background:**

Controlling cholesterol levels is one of the primary goals of preventing atherosclerotic plaque progression in patients undergoing coronary artery bypass graft (CABG) surgery. This study aimed to investigate the impact of serum cholesterol profile at multiple time points following isolated CABG surgery on long-term patient outcomes.

**Method:**

This retrospective cohort study was conducted on the admission and follow-up data of isolated CABG patients from the Tehran Heart Center registry between 2009 and 2016. The association of low-density lipoprotein (LDL), high-density lipoprotein (HDL), and their ratio as an atherogenic index with major adverse cardiac and cerebrovascular events (MACCE) and all-cause mortality were evaluated using time-varying survival analysis methods.

**Result:**

A total of 18657 patients were included in this analysis. After adjusting for known confounding factors, no significant difference in all-cause mortality and MACCE was observed at different LDL levels. The incidence of acute coronary syndrome (ACS) in patients with LDL > 100 mg/dl and LDL < 50 mg/dl was significantly higher than in the control group (*P*-value = 0.004 and 0.04, respectively). The incidence of cerebrovascular accidents (CVA) at LDL > 100 mg/dl was also significantly higher compared to the control group (*P* -value = 0.033). Lower HDL levels were significantly associated with a higher MACCE (*P* -value < 0.001), all-cause mortality (*P* -value < 0.001), ACS (*P* -value = 0.00), and CVA (*P* -value = 0.014). The atherogenic index was also directly related to MACCE and all its components (all *P*-values < 0.001).

**Conclusion:**

LDL/HDL ratio is suggested as a better marker for secondary prevention goals compared to LDL alone in patients undergoing CABG surgery.

## Introduction

Dyslipidemia, particularly elevated low-density lipoprotein cholesterol (LDL), is one of the major risk factors leading to the formation and progression of atherosclerotic plaques that lead to coronary artery disease (CAD) [1]. LDL particles can pass through the endothelium of the vascular wall, oxidize, and subsequently cause inflammation and damage to the vascular wall and surrounding smooth muscle cells [2]. These processes can finally lead to atherosclerosis and the subsequent events resulting from vascular stenosis and occlusion. Therefore, controlling the serum level of LDL is one of the primary goals in preventing atherosclerosis progression and its complications [3, 4].

Coronary artery bypass graft (CABG) surgery is a common treatment strategy for coronary artery disease. Over 800,000 CABG surgeries are performed annually in the world [5]. Several indices, including cholesterol profile, can exacerbate coronary artery atherosclerosis and lead to major adverse cardiac and cerebrovascular events (MACCE) after the surgery [5–7]. Secondary prevention of CAD following surgery and control of established risk factors, such as serum cholesterol profile, are required to obtain favorable outcomes. Given the dynamic nature of cholesterol profiles based on adherence to lipid-lowering medicines, conducting a time-varying analysis is critical in evaluating the impact of these risk variables on CABG patients' long-term results. This is especially critical because patient adherence to lipid-lowering medicines has significantly impacted patient outcomes [8, 9].

Previous studies have reported the benefits of high-intensity statin therapy after CABG surgery in reducing the incidence of MACCE [10–12]. The European Society of Cardiology (ESC) and the American Heart Association/American College of Cardiology (AHA/ACC) practice guidelines recommend serum LDL reduction as a protective measure against long-term MACCE [13, 14]. Nonetheless, the current literature includes sparse evidence comparing different levels of serum cholesterol components at multiple time points after isolated CABG surgery on the incidence of MACCE. On the other hand, several studies have suggested atherogenic indices, such as LDL/HDL ratio, as a risk predictor of cardiovascular disorders [15–18]. However, the prediction value of these indices for CABG outcomes has not yet been reported. Previous studies have mainly focused on comparing the effect of lipid-lowering agents and their doses on the outcomes of CABG surgery without assessing the impact of the atherogenic index over the follow-up time [19].

This study aimed to assess the impact of postoperative serum LDL and HDL levels at multiple time points on long-term patient outcomes of CABG surgery. Furthermore, the ratio of these factors was investigated as an atherogenic index at various time points after the procedure, considering the dynamic nature of their influence using a time-varying analysis.

## Methods

### Study design

This was a large-scale retrospective cohort study on patients undergoing isolated CABG surgery at Tehran Heart Center from March 2009 to March 2016. Data on these patients were retrieved from the Adult Cardiac Surgery Database [20, 21]. The proposal for this study was reviewed and approved by the research board and committee of ethics at Tehran Heart Center (IR.TUMS.MEDICINE.REC.1398.841).

### Study setting

Tehran Heart Center is a high-volume tertiary referral hospital affiliated with the Tehran University of Medical Sciences, operating since 2002.

### Participants

The study inclusion criteria consisted of all adult patients undergoing isolated CABG surgery between 2009–2016. Patients with simultaneous valvular or congenital heart defect surgery, missing data, or no follow-up data were excluded from the study. Informed consent was obtained from all patients at admission to allow the use of their clinical data for research purposes.

CABG surgeries were performed at Tehran Heart Center by a team of expert cardiovascular surgeons. Following CABG surgery, all patients are evaluated at the surgery follow-up clinic 4–6 and 12 months after surgery and annually thereafter. Patients' symptoms, changes in the risk factors, laboratory tests, and medications were reviewed at each visit. The laboratory tests included measurement of fasting blood glucose, urea, creatinine, lipid profile, complete blood cell count, and liver function tests. Any occurrence of MACCE, defined by non-fatal acute coronary syndrome (ACS), non-fatal revascularization, and non-fatal cerebrovascular accident (CVA), was also recorded. A general practitioner conducted follow-up visits in person, and a trained nurse attained the patient's symptoms, MACCE, and test results. If a patient could not attend their follow-up appointment, a trained nurse would complete the form over the phone. Additionally, the mortality rates of the patients were investigated by phone calls, and a mortality form was completed for every deceased patient to identify the cause of death, i.e., cardiac or non-cardiac.

According to the previous studies [22–25], Eligible patients were categorized into four groups according to the LDL levels at each visit: 1- LDL < 50 mg/dl, 2- LDL = 50–70 mg/dl, 3- LDL = 70–100 mg/dl, 4- LDL > 100 mg/dl. Based on the latest guidelines, LDL = 50–70 mg/dl was considered the reference group and other LDL levels were compared to this group. All baseline and follow-up variables were subsequently compared between the LDL groups to assess their predictive value for MACCE and all-cause mortality. Subsequently, the effect of HDL levels and atherogenic index (LDL/HDL ratio) on MACEE and its components was further evaluated.

### Statistical analysis

The normally distributed continuous variables were described as mean with standard deviation (SD) and were compared between the LDL level groups using one-way analysis of variance (ANOVA). The skew-distributed variables were expressed as median with 25^th^ and 75^th^ percentiles and were compared between groups applying the Kruskal–Wallis test. Categorical variables were described as frequency with percentages, and their distribution among LDL level groups was compared using a chi-square test. The unadjusted and adjusted effects of LDL and LDL/HDL ratio as a time-varying covariate on all-cause mortality and MACCE were evaluated using an extended COX regression model. The results were reported through hazard ratios (HR) and 95% confidence intervals (CIs). The proportional hazards (PH) assumption was assessed using the chi-square test of the correlation coefficient of scaled Schoenfeld residuals and time. The confounder variables for the adjusted model were selected according to the literature. The effects of LDL levels and LDL to HDL ratio on each MACCE component, including ACS and CVA, were assessed in a competing risk setting, considering death as a competing event. The effects were reported via sub-distribution hazard ratio (sHR) and 95% CI. Statistical analyses were conducted using Stata Statistical Software, release 15.2 (College Station, TX: Stata Corp.).

## Results

### Participants

A total of 21380 CABG surgeries were performed at Tehran Heart Center during the study period. After excluding patients according to the inclusion/exclusion criteria, 18941 patients who underwent isolated CABG surgery were eligible for the study. Follow-up data regarding 18657 of these patients were retrieved. The follow-up record for 284 patients (1.4%) could not be achieved (due to migrations, unwillingness for follow-up visits, or lack of a contact number). The median follow-up duration was 4.5 years, with 80754 patient-years of follow-up. A final number of 18657 patients were included in the study with an average age of 67.1 ± 9.7 years old. There were 13689 (73.4%) male patients in the study population.

### Descriptive data

**Fig. 1:**
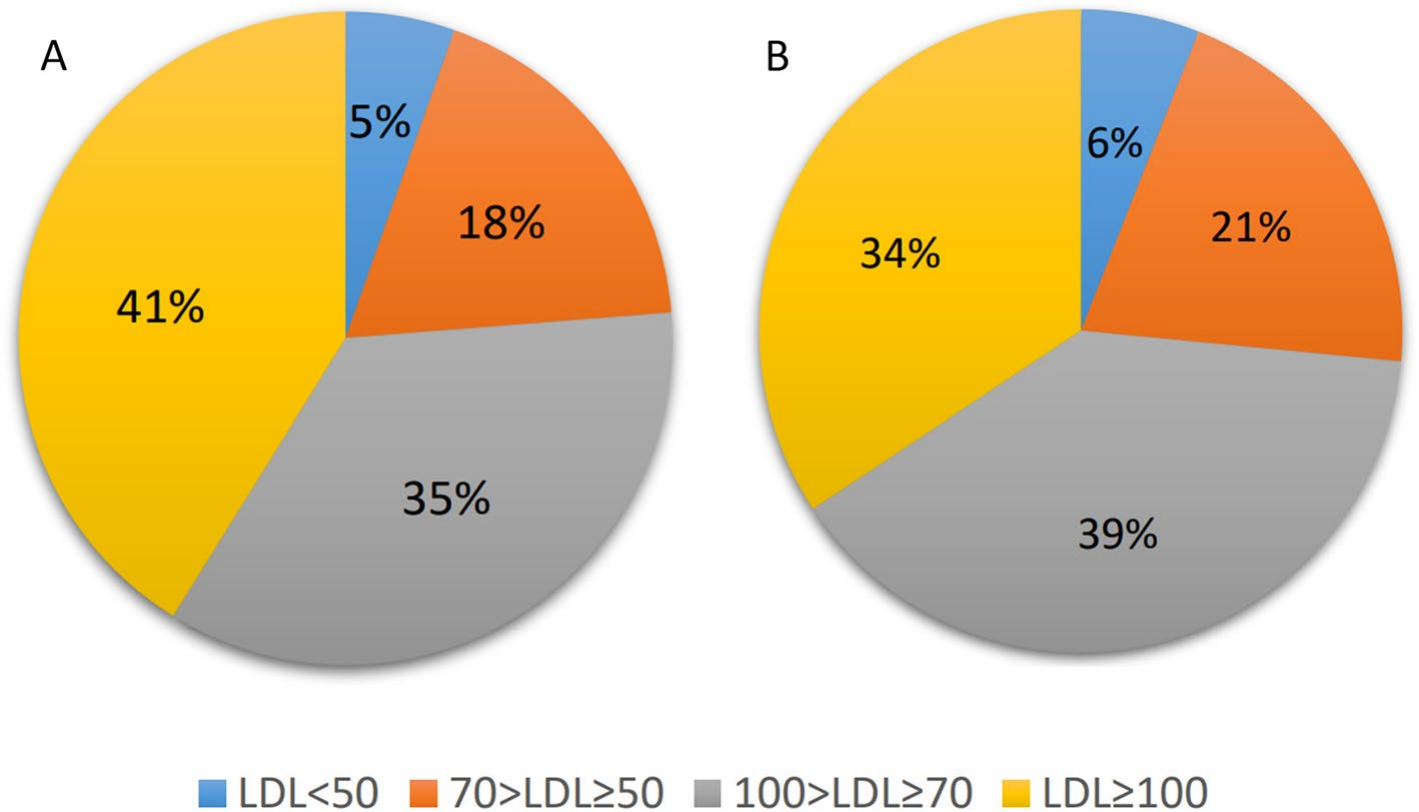
Distribution of serum LDL groups at the time of surgery (**A**) and at patient follow-up visits (**B**)

In a total of 82421 follow-up visits, postoperative LCL and HDL levels were measured and reported (an average of 4.4 measurements for every patient). According to the baseline LDL levels, patients were divided into four groups (LDL < 50, 50 < LDL < 70, 70 < LDL < 100, and LDL > 100). Figure 1A and B demonstrate the distribution of serum LDL groups at the time of surgery and follow-up visits, respectively.

Table 1 describes the patient characteristics for the total population and 4 LDL-level groups. Among the most common comorbidities, 39.9% of the patients were diagnosed with diabetes mellitus, 54.7% had hypertension, and 2.2% had chronic kidney disease. There was an 18.1% and 11% rate for cigarette and opium addiction at the time of surgery, respectively.

**Table 1:** Baseline demographics of patients undergoing isolated CABG based on serum LDL level

	**Total (*n* = 18,657)**	**LDL < 50 (*n* = 1,009)**	**50** $\le \mathbf{L}\mathbf{D}\mathbf{L}-\mathbf{C}<70$** (*n* = 3,423)**	**70** $\le \mathbf{L}\mathbf{D}\mathbf{L}-\mathbf{C}<100$** (*n* = 6,547)**	**100** $\le \mathbf{L}\mathbf{D}\mathbf{L}-\mathbf{C}$** (*n* = 7,678)**	***P*-value**
Age (years)^b^	9.7 $\pm$ 67.1	9.5 $\pm$ 67.9	9.7 $\pm$ 67.5	9.6 $\pm$ 67.1	9.9 $\pm$ 66.7	< 0.001
Male gender (%)^b^	13689(73.4)	839(83.2)	2736(79.9)	4828(73.7)	5286(68.8)	< 0.001
BMI (kg/${\mathrm{m}}^{2})$ ^b^	4.2 $\pm$ 27.2	4.1 $\pm$ 26.8	4.0 $\pm$ 26.9	4.3 $\pm$ 27.3	4.2 $\pm$ 27.4	< 0.001
EF (%)^b^	9.1 $\pm$ 46.1	9.1 $\pm$ 45.6	9.3 $\pm$ 45.4	9.1 $\pm$ 45.8	9.1 $\pm$ 46.6	< 0.001
SBP (mmHg)^b^	22.5 $\pm$ 146.3	22 $\pm$ 145.1	23.2 $\pm$ 147.4	22.2 $\pm$ 145.9	22.6 $\pm$ 146.3	0.13
DBP (mmHg)^b^	9.8 $\pm$ 78.3	9.7 $\pm$ 78.1	10.6 $\pm$ 78.9	9.4 $\pm$ 78.0	9.8 $\pm$ 78.3	0.06
DM (%)	7442(39.9)	545(54.0)	1500(43.8)	2623(40.1)	2744(36.1)	< 0.001
HTN (%)	10066(54.7)	581(57.6)	1884(55.0)	3577(54.6)	4024(52.4)	< 0.001
CKD (%)	403(2.2)	43(4.3)	104(3.1)	135(2.1)	121(1.6)	< 0.001
Family history (%)	6761(36.2)	342(32.1)	1223(35.7)	2350(35.9)	2864 (37.3)	0.008
COPD (%)	652(3.5)	35(3.5)	114(3.4)	243(3.7)	260(3.4)	0.69
CVA (%)	1252(6.8)	91(9.1)	254(7.5)	445(6.9)	462(6.1)	< 0.001
Cigarette smoker (%)	3371(18.1)	168(16.7)	582(17.0)	1170(17.9)	1451(18.9)	< 0.001
Opium user (%)	2056(11.0)	135(13.4)	400(11.7)	741(11.3)	780(10.2)	< 0.001
Previous PCI (%)	825(4.4)	54(5.4)	159(4.7)	302(4.6)	310(4.1)	0.13
MI within 7 days (%)	1635(8.8)	54(5.4)	178(5.2)	509(7.8)	894(11.7)	< 0.001
Off-pump surgery (%)	1558(8.7)	109(11.3)	347(10.3)	589(9.1)	513(7.2)	< 0.001
Cross clamp time (min)^a^	39(30–50)	40(31–49)	39(30–50)	39(30–49)	39(30–50)	0.044
Perfusion time (min)^a^	67(55–84)	68(55–81)	66(54–82)	67(54–83)	68(55–85)	0.047
Intubation time (hr)^a^	9.5(7.5–12.5)	9.5(7.4–12.5)	10.0(7.5–13.0)	10.0(7.5–12.5)	9.5(7.5–12.5)	0.02
Graft number (N)^a^	3(3–4)	4(3–4)	3(3–4)	3(3–4)	3(3–4)	0.11
ICU time (hr)^a^	29.0(23.0–65.5)	28.0(23.0–59.3)	27.5(23.0–53.5)	28.5(23.0–65.0)	39.0(23.0–67.0)	0.019

*LDL (mg/dl)* Low-density lipoprotein cholesterol, *BMI* Body mass index, *EF* Ejection fraction, *SBP* Systolic blood pressure, *DBP* Diastolic blood pressure, *DM* Diabetes, *HTN* Hypertension, *CKD* Chronic kidney disease, *COPD* Chronic obstructive pulmonary disease, *CVA* Cerebrovascular accident, *PCI* Percutaneous intervention, *ICU* Intensive care unit

^a^Reported as Median (Interquartile range)

^b^Reported as Mean ± Standard deviation

In order to identify cofounder factors, 16 variables were selected based on the differences observed between variables in four LDL groups. The variables identified as confounder factors were age, sex, body mass index, ejection fraction (EF), blood pressure, diabetes, smoking, opium, kidney failure, chronic obstructive pulmonary disease (COPD), CVA, serum HDL level, number of grafts, Intensive care unit (ICU) stay, myocardial infarction in the last seven days, and off-pump CABG surgery.

### Outcome data

The primary outcome variables of all-cause mortality, MACCE, CVA, and ACS in the follow-up period are compared between the four LDL level groups in Table 2 before the adjustments for confounder variables. The incidence of all-cause mortality in the 70 ≤ LDL < 100 mg/dl (*P* -value = 0.025) and LDL ≥ 100 mg/dl (*P* -value < 0.001) groups and MACCE in the LDL ≥ 100 mg/dl group (*P* -value < 0.001), was significantly less than the reference group 50 < LDL ≤ 70 mg/dl (Table 2).

**Table 2:** Univariate COX regression for MACCE and all-cause mortality in various serum LDL levels with 50 ≤ LDL-C < 70 as the reference group

	**All-cause mortality**		**MACCE**		**ACS**		**CVA**	
	**HR(CI)**	***P*-value**	**HR(CI)**	***P*-value**	**sHR(CI)**	***P*-value**	**sHR(CI)**	***P*-value**
**LDL-C < 50 mg/dl**	1.07 (0.88–1.29)	0.507	1.11 (0.95–1.28)	0.181	1.24 (0.97–1.58)	0.091	1.20 (0.78–1.85)	0.413
**70 ≤ LDL-C < 100 mg/dl**	0.87 (0.78–0.98)	0.025	0.96 (0.88–1.05)	0.352	1.11 (0.95–1.29)	0.196	0.97 (0.74–1.28)	0.839
**LDL-C ≥ 100 mg/dl**	0.70 (0.62–0.78)	< 0.001	0.84 (0.76–0.91)	< 0.001	1.07 (0.92–1.24)	0.37	0.98 (0.75–1.29)	0.907

*LDL-C (mg/dl)* Low-density lipoprotein cholesterol, *MACCE* Major adverse cardiovascular and cerebrovascular event

**Fig. 2:**
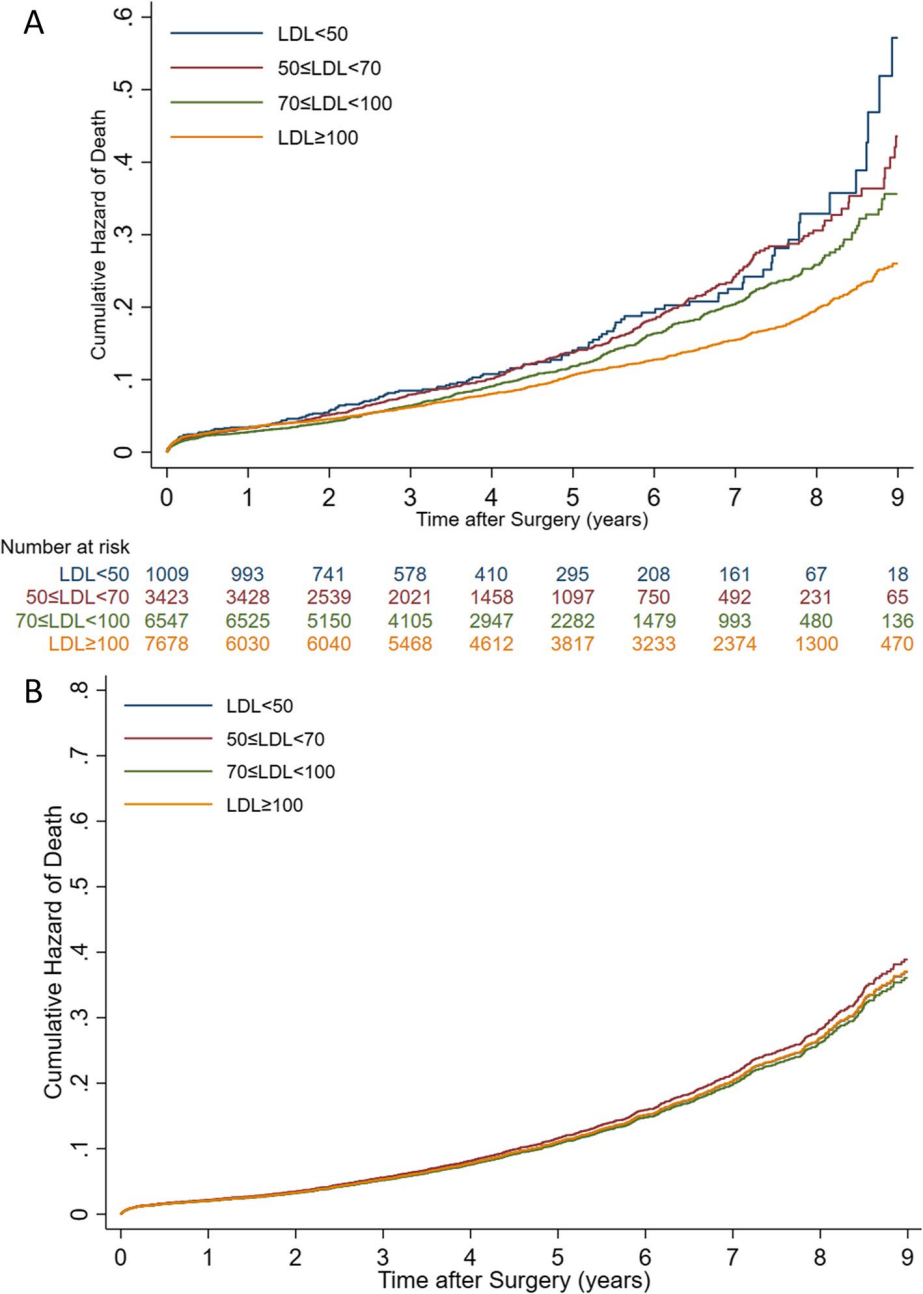
Correlation between different groups of LDL and all-cause mortality before (**A**) and after adjustment for confounding factors (**B**). The Proportional Hazards (PH) assumption was not rejected in the final model (PH test *P*-value = 0.190)

**Fig. 3:**
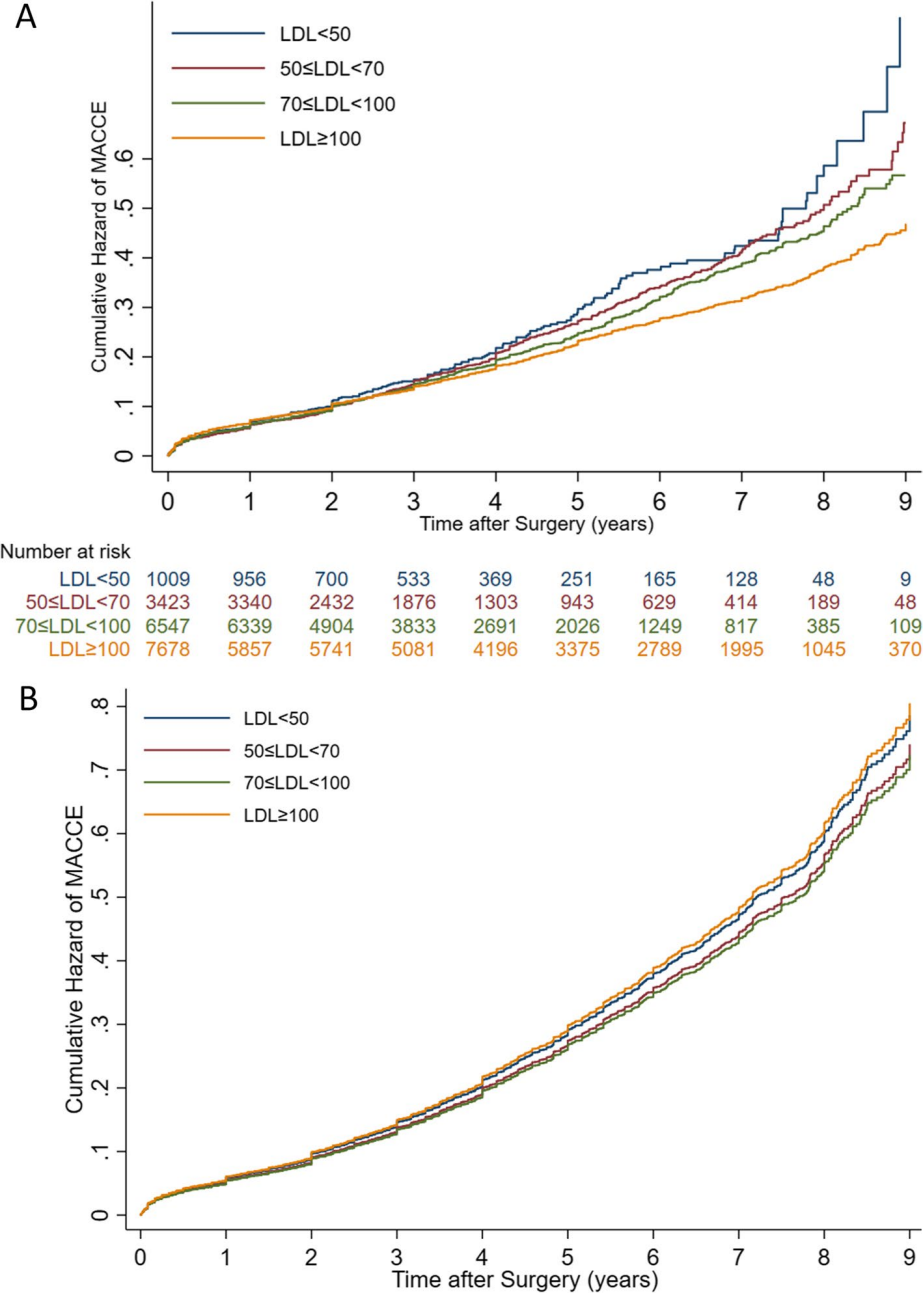
Correlation between different groups of LDL and MACCE before (**A**) and after adjustment for confounding factors (**B**). The Proportional Hazards (PH) assumption was not rejected in the final model (PH test *P*-value = 0.461)

Table 3 compares the primary outcome variables between four LDL groups after adjustment for 16 confounder variables. The relationship between four LDL groups and primary outcome variables (all-cause mortality and MACCE) before and after adjustment for confounding factors is illustrated in Figs. 2 and 3.

**Table 3:** Multivariable results for MACCE, all-cause mortality, ACS, and CVA for LDL, HDL, and LDL/HDL

	**All-cause mortality**		**MACCE**		**ACS**		**CVA**	
	**HR(CI)**	***P*-value**	**HR(CI)**	***P*-value**	**sHR(CI)**	***P*-value**	**sHR(CI)**	***P*-value**
**LDL < 50**	0.95 (0.77–1.17)	0.625	1.06 (0.9–1.24)	0.490	1.31 (1.01–1.69)	0.04	1.21 (0.76–1.93)	0.414
**70** $\le {\varvec{L}}{\varvec{D}}{\varvec{L}}-{\varvec{C}}<100$	0.93 (0.82–1.05)	0.227	0.97 (0.88–1.07)	0.541	1.07 (0.91–1.26)	0.385	1.03 (0.77–1.37)	0.861
**100 ** $\le {\varvec{L}}{\varvec{D}}{\varvec{L}}$**-C**	0.95 (0.84–1.08)	0.464	1.07 (0.97–1.18)	0.152	1.26 (1.08–1.48)	0.004	1.36 (1.03–1.81)	0.033
**HDL**	0.98 (0.98–0.99)	< 0.001	0.99 (0.98–0.99)	< 0.001	0.99 (0.98–1)	0.004	0.99 (0.97–1)	0.017
**LDL/HDL**	1.10 (1.06–1.15)	< 0.001	1.11 (1.08–1.15)	< 0.001	1.121 (1.07–1.18)	< 0.001	1.19 (1.09–1.30)	< 0.001

*LDL (mg/dl)* Low-density lipoprotein cholesterol, *HDL* High-density lipoprotein cholesterol, *MACCE* Major adverse cardiovascular and cerebrovascular event, *ACS* Acute coronary syndrome, *CVA* Cerebrovascular event

According to this adjusted model, all-cause mortality and MACCE incidence in any LDL group were not significantly different from the reference group (Table 3). However, the incidence of ACS in patients with LDL levels greater than 100 mg/dl and less than 50 mg/dl during follow-up was significantly higher than in the reference group (*P*-value = 0.004 and *P*-value = 0.04, respectively) after adjustment for known confounding factors. Furthermore, CVA was significantly higher in cases with LDL levels greater than 100 mg/dl compared to the reference group (*P*-value = 0.033, Table 3).

Moreover, a lower HDL level was significantly associated with a higher incidence of MACCE (*P*-value < 0.001), all-cause mortality (*P*-value < 0.001), ACS (*P*-value = 0.004), and CVA (*P*-value = 0.017). Regression analysis revealed that with every 1 mg/dl increase in serum HDL level, the incidence of all-cause mortality decreases by 7.1% and MACCE by 4.1%.

**Fig. 4:**
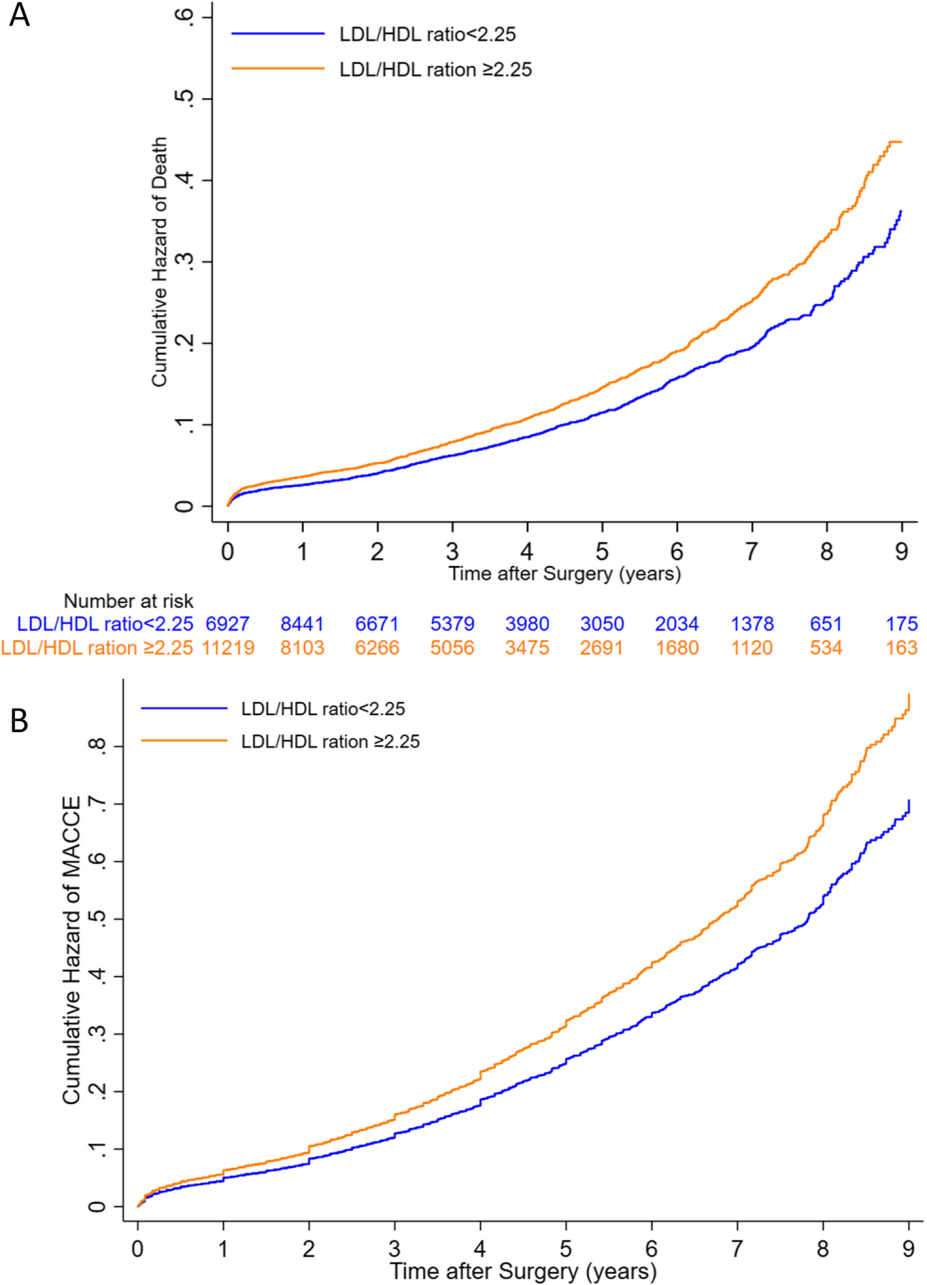
Comparison of long term all-cause mortality for patients above and below the suggested cut-off point for LDL/HDL ratio (2.25) before (**A**) and after adjustment (**B**) for confounding factors. The Proportional Hazards (PH) assumption was not rejected in the final model (PH test *P*-value = 0.756)

**Fig. 5:**
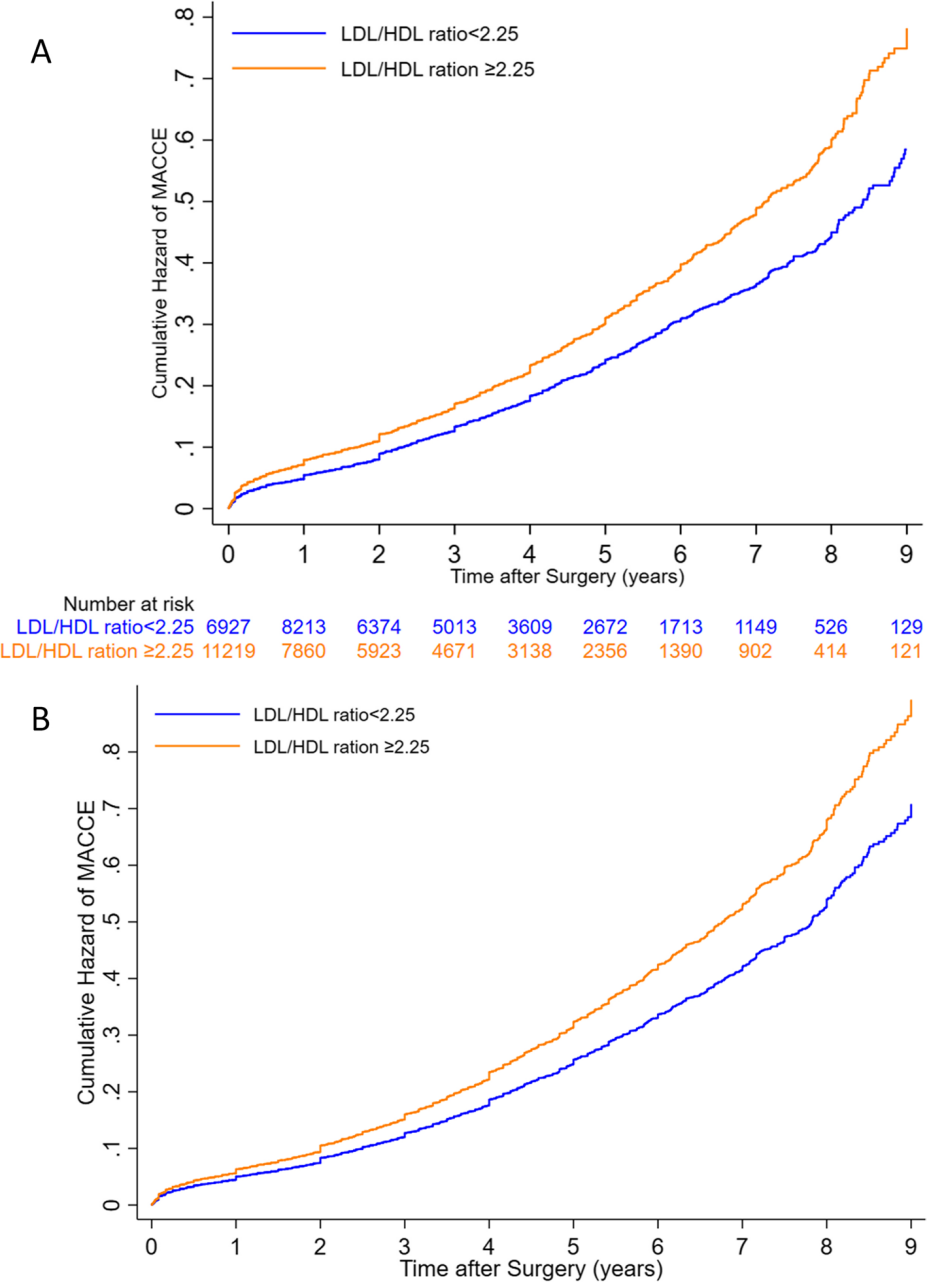
Comparison of long-term MACCE for patients above and below the suggested cut-off point for LDL/HDL ratio (2.25) before (**A**) and after adjustment (**B**) for confounding factors. The Proportional Hazards (PH) assumption was not rejected in the final model (PH test *P*-value = 0.477)

Finally, a higher LDL/HDL ratio was significantly associated with increased incidence of MACCE, all-cause mortality, ACS, and CVA (all *P*-values < 0.001). The regression analysis results suggested that with every 0.5 unit increase in this ratio, all-cause mortality increased by 5%, MACCE by 5.7%, ACS by 6%, and CVA by 9.5%. By selecting the median for the LDL/HDL ratio (2.25) as a cut-off point, the patients with a higher than median LDL/HDL value had a significantly higher HR for MACCE rate (26%), the all-cause mortality rate (23%), ACS (26%), and CVA (37%). Long-term survival analysis comparison between 2 groups according to LDL/HDL ratio is demonstrated in Figs. 4 and 5 indicating a significantly higher long-term mortality and MACCE in patients with an LDL/HDL ratio of higher than 2.25. The Proportional Hazards (PH) assumption was not violated in any of the final models for different patient groups according to LDL levels or LDL/HDL ratio.

Moreover, After the adjustments for known confounding factors, older age, male gender, lower EF, hypertension, renal failure, COPD, CVA, lower HDL, longer ICU hospitalization, and off-pump surgery were independently associated with a significantly higher MACCE and mortality rate after CABG (all *P-value*s < 0.001, Table 4).

**Table 4:** Multivariable COX regression for all-cause mortality and MACCE

	**All-cause mortality**		**MACCE**	
**Variable**	**HR(CI)**	***P*-value**	**HR(CI)**	***P*-value**
Age	1.05(1.04–1.05)	< 0.001	1.02(1.02–1.03)	< 0.001
Male gender	0.72(0.64–0.8)	< 0.001	0.7(0.64–0.76)	< 0.001
BMI	1(0.98–1.01)	0.477	1(1–1.01)	0.32
EF	0.96(0.95–0.96)	< 0.001	0.97(0.97–0.98)	< 0.001
HTN	0.74(0.66–0.84)	< 0.001	0.71(0.65–0.77)	< 0.001
Renal failure	3.7(3.08–4.43)	< 0.001	2.5(2.12–2.94)	< 0.001
COPD	1.67(1.37–2.04)	< 0.001	1.33(1.13–1.56)	< 0.001
CVA	1.86(1.6–2.16)	< 0.001	1.69(1.51–1.91)	< 0.001
Current cigarette user	–––-	–––-	1.28(1.14–1.44)	< 0.001
Current Opium user	1.38(1.2–1.59)	< 0.001	1.07(0.95–1.2)	0.241
HDL	0.983	< 0.001	0.986	< 0.001
MI within 7 days	1.06(0.9–1.24)	0.497	1.08(0.96–1.22)	0.176
Off-pump surgery	1.51(1.25–1.83)	< 0.001	1.2(1.04–1.39)	0.015
Graft number	0.96(0.9–1.01)	0.126	0.96(0.92–1)	0.068
ICU time	1(1–1)	< 0.001	1(1–1)	< 0.001
Previous PCI	–––-	–––-	1.34(1.14–1.57)	< 0.001

*BMI* Body mass index, *EF* Ejection fraction, *HTN* Hypertension, *COPD* Chronic obstructive pulmonary disease, *CVA* Cerebrovascular accident, *HDL* High-density lipoprotein cholesterol, *MI* Myocardial infarction, *ICU* Intensive care unit, *PCI* Percutaneous coronary intervention

## Discussion

This study evaluated the effect of cholesterol profile at multiple follow-up time points on long-term outcomes of isolated CABG patients in a sizeable registry-based cohort study utilizing a time-varying analysis. The incidence of ACS was significantly higher in patients with LDL levels of more than 100 mg/dl and less than 50 mg/dl, although all-cause mortality and MACCE were not associated with different LDL levels. In contrast, HDL levels were significantly associated with postoperative MACCE and its components. Furthermore, a strong association was detected between the atherogenic index and all cardiovascular outcomes (MACCE, ACS, CVA, and all-cause mortality) following CABG surgery.

The current study results align with the findings of the CASCADE study, which suggests that reaching LDL levels of less than 100 mg/dl with statin therapy is independently associated with greater patency of coronary arteries following CABG. This study also reported that lower LDL (< 70 mg/dl) levels had no further improvement in the patency of coronary arteries [11].

A paradoxical increase in the incidence of post-CABG ACS in the LDL < 50 mg/dl group was also detected. Several recent studies have also detected a similar paradoxical cardiovascular outcome in patients with low LDL levels [26–29]. In a study by Nakahashi et al., all-cause mortality rates were paradoxically higher for patients with ACS when their LDL levels were below 100 mg/dl [26]. One possible reason could be the lower dose of post-discharge statin treatment received by patients with LDL < 100 mg/dl compared to patients with LDL levels above 100 mg/dl. Statins are essential in protecting the coronary endothelium due to their anti-inflammatory effects and stabilization of atherosclerotic plaques [30]. Furthermore, Wang et al., in a study on 41,229 CAD patients, reported that malnutrition might be the cause of poor cardiovascular outcomes in patients with a low LDL level [28]. Additional studies are required to fully understand the impact of low LDL levels and statin treatments on the outcomes of CAD patients.

Another important finding of this study is the association of HDL levels with long-term CABG outcomes. Previous studies have also reported this inverse relationship between serum HDL and cardiovascular events in CAD patients [31]. In a survey by Domanski et al. on 1,248 CABG patients, the survival rate was significantly higher in patients with HDL > 35 mg/dl compared to patients with HDL < 35mg/dl. Similarly, patients with a higher HDL reported significantly lower post-CABG revascularization and myocardial infarction [6]. Several studies have indicated that although increasing serum HDL levels through non-pharmacological measures (diet, exercise, and smoking cessation) is associated with better cardiovascular outcomes [32–34], increasing HDL levels through medication was not effective in reducing cardiovascular events [31, 35, 36].

Lastly, LDL/ HDL ratio was investigated as an atherogenic index to simultaneously assess the effects of HDL and LDL levels on patient outcomes, revealing a significant association. Recent studies also propose this index as a valuable predictor for cardiovascular risk assessments [37–40]. In a study on the Framingham data, Nam et al. reported HDL/Total cholesterol and HDL/LDL as better predictors for cardiovascular outcomes compared to total cholesterol or LDL alone [41]. In an assessment of the Finnish Kuopio Ischemic heart disease (IHD) prospective cohort study, the LDL/HDL ratio was an independent risk factor for sudden cardiac death in IHD patients [42]. Also, Sheng et al. suggested a non-high-density/HDL ratio as a better predictor of diabetes risk compared to cholesterol components [43]. Considering emerging suggestions on utilizing atherogenic index as a cardiovascular predictive factor, LDL/HDL ratio can be a practical outcome predictor for CABG patients.

Nonetheless, more studies are required to reach an exact LDL/HDL ratio threshold as a post-CABG patient management goal. According to the current study, extended COX regression could not determine a specific off-cut point for the atherogenic index that best predicts a patient's cardiovascular outcomes. Therefore, the median of this ratio, which measured 2.25, can be regarded as a secondary objective after attaining LDL levels below 100 mg/dl.

The large sample size, long-term patient follow-ups, multiple cholesterol profile assessments at different time points, and time-varying analysis can distinguish the present study from the current evidence in the literature. The majority of previous studies, including the ACTIVE trial, examined the effect of different therapeutic doses of statins on anatomical outcomes (e.g., coronary artery occlusion in coronary computed tomography angiography) [10]. However, the assessed study endpoint was MACCE, its components, and all-cause mortality. The report of these outcomes can be more practical in predicting long-term surgical results as postoperative artery occlusion depends on multiple variables, such as surgical techniques.

### Strengths and limitations

The large patient population and cholesterol profile assessments at multiple time points were the key strengths of this study. Nevertheless, the main limitation of the present study was the absence of statin treatment dose in the results. Changes in the statin dose based on patients' LDL levels during follow-up could distort the study's results. It was not possible to incorporate additional atherogenic indices like APO-A, APO-B, and LP(a) into the study since these measurements were not part of the routine assessment for the CABG patients. Lastly, despite attempts to identify all variables influencing the cardiovascular outcomes of patients following CABG surgery for adjusting confounding factors, other factors could be impacting the study results.

## Conclusion

According to the study findings, LDL level assessment as secondary prevention is insufficient to guide the treatment of CABG patients. Physicians should consider LDL/HDL ratio to better predict patients' cardiovascular risk. A recommended HDL/LDL ratio of < 2.25 and LDL < 100 mg/dl is advised as a secondary prevention goal for patients after isolated CABG surgery and can significantly improve long-term patient outcomes.

## Data Availability

The datasets regarding the current study are available from the corresponding author upon reasonable request.

## Abbreviations

ACS: Acute Coronary Syndrome
CABG: Coronary Artery Bypass Graft
CAD: Coronary artery disease
CI: Confidence Interval
CKD: Chronic kidney disease
COPD: Chronic Obstructive Pulmonary Disease
CVA: Cerebrovascular Accident
DBP: Diastolic blood pressure
DM: Diabetes Mellitus
EF: Ejection Fraction
ESC: European Society of Cardiology
HDL: High-density Cholesterol
HTN: Hypertension
HR: Hazard Ratio
ICU: Intensive Care Unit
LDL: Low-density Cholesterol
MACE: Major Adverse Cardiovascular Events
MI: Myocardial Infarction
PCI: Percutaneous Intervention
SBP: Systolic blood pressure
